# Sarcoma-Targeting Peptide-Decorated Polypeptide Nanogel Intracellularly Delivers Shikonin for Upregulated Osteosarcoma Necroptosis and Diminished Pulmonary Metastasis: Erratum

**DOI:** 10.7150/thno.46662

**Published:** 2020-04-12

**Authors:** Suoyuan Li, Tao Zhang, Weiguo Xu, Jianxun Ding, Fei Yin, Jing Xu, Wei Sun, Hongsheng Wang, Mengxiong Sun, Zhengdong Cai, Yingqi Hua

**Affiliations:** 1 Department of Orthopedics, Shanghai General Hospital, Shanghai Jiao Tong University School of Medicine, Shanghai 200080, P. R. China; 2 Key Laboratory of Polymer Ecomaterials, Changchun Institute of Applied Chemistry, Chinese Academy of Sciences, Changchun 130022, P. R. China; 3 Shanghai Bone Tumor Institution, Shanghai 201620, P. R. China; 4 Department of Orthopedics, Suzhou Municipal Hospital, Nanjing Medical University, Suzhou 215000, P. R. China

In the initially published version of this article 1, the marker “E” was missed in Figure 3E, the Western blot bands were incorrectly displayed in Figure 5F, and the values on the ordinate were incorrectly labeled in Figure 6C.

The corrected Figure 3E, Figure 5F, and Figure 6C are as follows:

The corrections made in this erratum do not affect the original conclusions. The authors apologize for any inconvenience or misunderstanding that these errors may have caused.

## Figures and Tables

**Figure 3:**
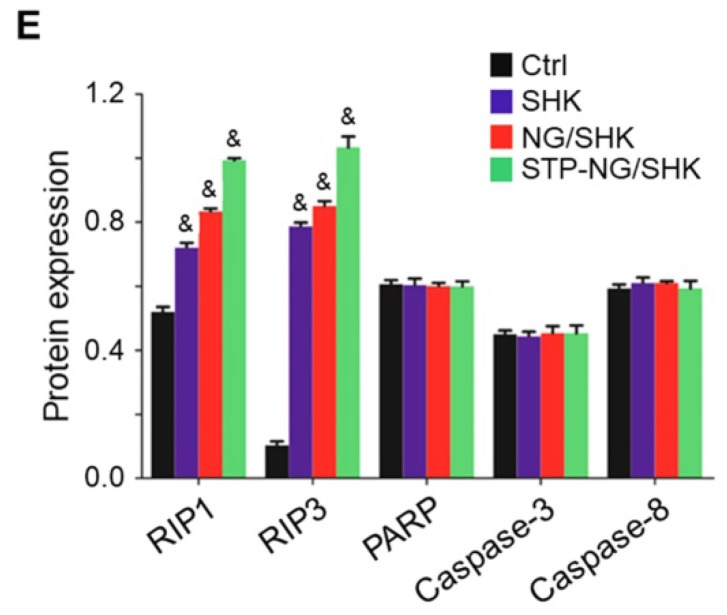
(E) Semiquantitative analysis of Western blot bands RIP1, RIP3, PARP, caspase-3, and caspase-8.

**Figure 5:**
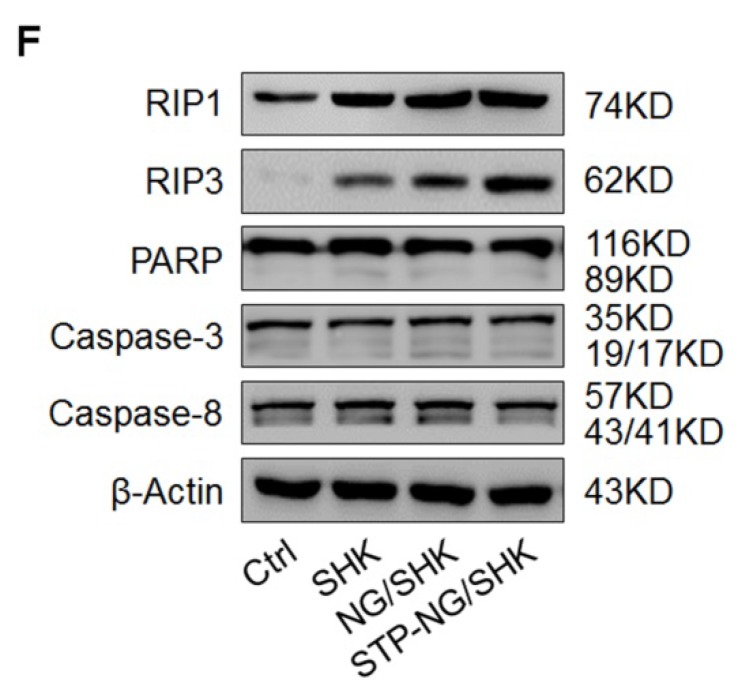
(F) RIP1, RIP3, PARP, caspase-3, and caspase-8 expression in primary tumor tissues per group detected by Western blot.

**Figure 6:**
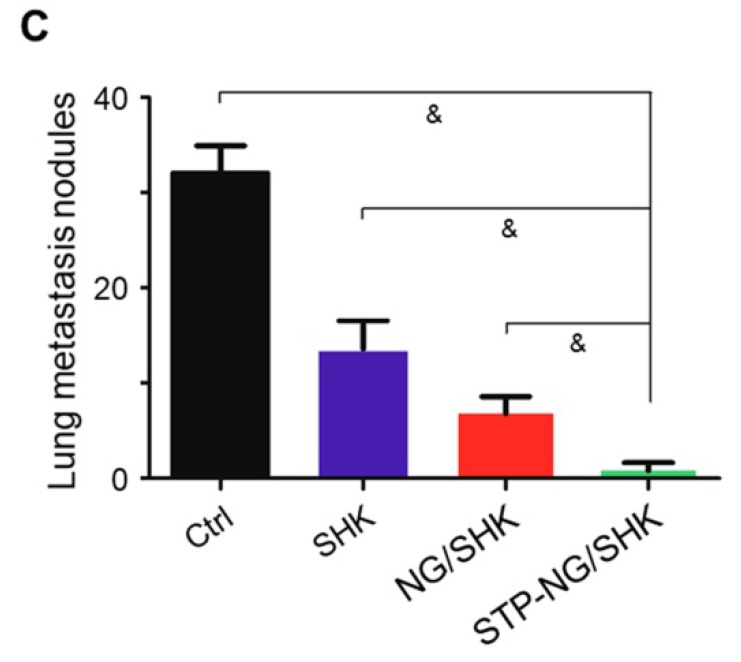
(C) Average microscopically counted lung metastases from largest coronal sections.
